# Errata

**Published:** 2015-06-12

## Vol. 64, No. 18

In the report, “Norovirus Outbreak Associated with a Natural Lake Used for Recreation — Oregon, 2014,” on pages 485–6, an error occurred in the fifth sentence of the first paragraph of the report. That sentence should read, “Analyses from a retrospective cohort study revealed that swimming at Blue Lake during July 12–13 was significantly associated with illness during July 13–14 (adjusted relative risk = 2.3; 95% confidence interval [CI] = 1.1–**4.9**).”

In the report, “Initiation of a Ring Approach to Infection Prevention and Control at Non-Ebola Health Care Facilities — Liberia, January–February 2015,” on page 505, an error occurred in the fifth sentence of the second paragraph. That sentence should read, “For three **of six** patients with available data, a fever (defined as a temperature >100.4°F [>38°C] taken with an infrared thermometer) was not recorded on arrival at the HCF.”

